# Effect of contact number among graphene nanosheets on the conductivities of tunnels and polymer composites

**DOI:** 10.1038/s41598-023-36669-1

**Published:** 2023-06-12

**Authors:** Yasser Zare, Tae-Hoon Kim, Nima Gharib, Young-Wook Chang

**Affiliations:** 1 Biomaterials and Tissue Engineering Research Group, Department of Interdisciplinary Technologies, Breast Cancer Research Center, Motamed Cancer Institute, ACECR, Tehran, Iran; 2 Department of Materials Science & Chemical Engineering, BK21 FOUR ERICA-ACE Center, Hanyang University ERICA, Ansan, 15588 Korea; 3 College of Engineering and Technology, American University of the Middle East, 54200 Egaila, Kuwait

**Keywords:** Engineering, Materials science

## Abstract

Simple equations are expressed for tunnel conductivity, tunnel resistance and conductivity of a graphene-filled composite by the number of contacts and interphase part. More specially, the active filler amount is suggested by interphase depth, which changes the contact number. The conductivity of nanocomposite is presented by filler content, filler dimensions, tunneling length and interphase depth. The innovative model is surveyed by the experimented conductivity of real examples. Too, the impacts of numerous issues on the tunnel resistance, tunnel conductivity and conductivity of nanocomposite are discussed to validate the novel equations. The estimates agree with the experimented data and the impacts of several terms on the tunnel resistance, tunnel conductivity and conductivity of system are sensible. Thin and big nanosheets positively affect the nanocomposite’s conductivity, but thick nanosheets improve the tunnel conductivity. High conductivity is found at short tunnels, while the nanocomposite’s conductivity directly depends on the tunneling length. The dissimilar effects of these features on the tunneling properties and conductivity are described.

## Introduction

The extraordinary aspect ratio (ratio of length to thickness) and immense surface area of graphene cause very low percolation onset in nanocomposites, which causes a significant conductivity and good rigidity by slight amount of graphene^1–14^. However, gaining a high-quality graphene and the homogenous dispersal of nanosheets in nanocomposite are two challenging tasks^4,15–19^. Polymer graphene nanocomposite commonly shows lower percolation onset and higher electrical conductivity compared to CNT nanocomposite^20^, but few incidences such as agglomeration, crimping and tough networking of graphene nanoparticles may weaken their efficiencies^21^.

Many parameters associated to graphene nanosheets can control the nanocomposite’s electrical conductivity like amount, conduction, size and dispersion quality^22–24^. The effects of these factors on the conductivity can be regarded by the modeling practices. Many models for conductivity of CNT products assume various parameters such as filler–polymer interface energy, tunneling mechanism, agglomeration and twistiness of CNT^25–28^. They can help to control the active factors on the conductivity. However, the suggested models for the conductivity of graphene–polymer composites (summarized as conductivity in the current paper) are restricted. The conductivity was widely assessed by conventional power-law equation^29–31^ using filler conduction, filler amount and percolation onset, but this model cannot show the novel features of nano-scale in the conductivity such as tunneling mechanism and interphase districts.

The electrical conductivity is mainly governed by charge tunneling as the current of electrons among adjacent nanosheets through tunneling mechanism^6,7,32–36^. Actually, the nanoparticles can contribute to the conductivity even once they are detached by short distance. In addition, the interphase parts about filler, owing to the large superficial area of nanoparticles change the properties of nanocomposite^37–42^. The impacts of interphase features on the rigidity of nanocomposites were investigated in the forgoing articles^43–45^. Likewise, the interphase parts can build the networks before the bodily assembly of particles^46–49^. Accordingly, the interphase parts can hasten the percolation onset and affect the magnitude of conductive networks. However, the effect of interphase on the conductivity was limitedly studied. Conclusively, several factors for tunnels and interphase affect the conductivity, while few models in this area have not properly reflected the impacts of these terms.

In this paper, simple equations are expressed for the tunnel conductivity, tunnel resistance and whole conductivity by the interphase parts. Specifically, the active filler amount is suggested by the interphase depth, which changes the number of contacts between nanosheets. As a result, filler share, filler dimensions, tunneling length and interphase depth control the conductivity. The model for conductivity is verified by the experimental results and also, the values of tunneling properties and interphase depth are determined. Also, an equation is developed for percolation onset to get the interphase deepness and tunneling length in the examples. Besides, the impressions of numerous issues on the tunnel resistance, tunnel conductivity and conductivity are investigated to justify the developed equations. The author hopes that the suggested model can attract the attentions of researchers in the future studies on polymer graphene nanocomposites to replace the conventional and incomplete models in this area.

## Development of equations

Electron tunneling extremely rely on the space among close nanoparticles as tunneling length^50,51^. Actually, charge can be conveyed in the tunnels, when the departure distance is sufficiently short. Polymer nanocomposite contains many tunneling spaces. So, the specifications of tunnels control the electrical conductivity.

Allaoui et al.^51^ submitted an equation for conductivity based on tunneling mechanism as:

1 $$\sigma = \frac{{nd^{2} }}{{R_{t} }}$$

where “*n*” is the quantity of contacts per unit volume, “*d*” is tunneling length between adjacent nanosheets and “*R*_*t*_” is intrinsic tunnel resistance.

“*n*” was suggested for three dimensional (3D) random fiber networks^51^ as:

2 $$n = \frac{{4\phi_{f}^{2} }}{{\pi a^{3} }}$$

where “$\phi_{f}$” is filler volume share and “*a*” is the diameter of CNT. Since both CNT and graphene have high aspect ratio, this equation can be applied for graphene networks by replacing of CNT diameter with graphene thickness as:

3 $$n = \frac{{4\phi_{f}^{2} }}{{\pi t^{3} }}$$

where “*t*” denotes the graphene thickness.

“*R*_*t*_” comprises the resistances of polymer film and nanosheets in the tunneling spaces, but the resistance of nanosheets is much smaller than that of polymer film. Therefore, “*R*_*t*_” is defined as:

4 $$R_{t} = \rho \frac{d}{Dt}$$

where “*ρ*” is tunnel resistivity and “*D*” is graphene length/width. As stated, “*t*” signifies the thickness of nanosheets and “*d*” is tunneling length between adjacent nanosheets.

It is possible to define the conductance of tunneling spaces by inverse “*R*_*t*_” as:

5 $$\sigma_{t} = \frac{Dt}{{\rho d}}$$

By assuming of Eqs. (3 and 4) into Eq. (1), the tunnel conductivity is suggested as:

6 $$\sigma = \frac{{4\phi_{f}^{2} Dd}}{{\rho \pi t^{2} }}$$

which relates the tunnel conductivity to the graphene amount, graphene dimensions as well as tunneling length and resistivity.

However, the interphase parts around graphene nanosheets can contribute to the networks and promote the productivity of nanoparticles in the nanocomposite.

The interphase volume share in the graphene sample^52^ is acquired by:

7 $$\phi_{i} = \phi_{f} \left( {\frac{{2t_{i} }}{t}} \right)$$

in which “*t*_*i*_” is the interphase deepness.

The active amount of graphene comprises the filler and interphase volume shares as:

8 $$\phi_{eff} = \phi_{f} + \phi_{i} = \phi_{f} \left( {1 + \frac{{2t_{i} }}{t}} \right)$$

So, “*n*” expressed in Eq. (3) is advanced to:

9 $$n = \frac{{4\phi_{eff}^{2} }}{{\pi t^{3} }} = \frac{{4\phi_{f}^{2} \left( {1 + \frac{{2t_{i} }}{t}} \right)^{2} }}{{\pi t^{3} }}$$

to accept the role of interphase parts in the number of contacts per volume.

**Figure 1:**
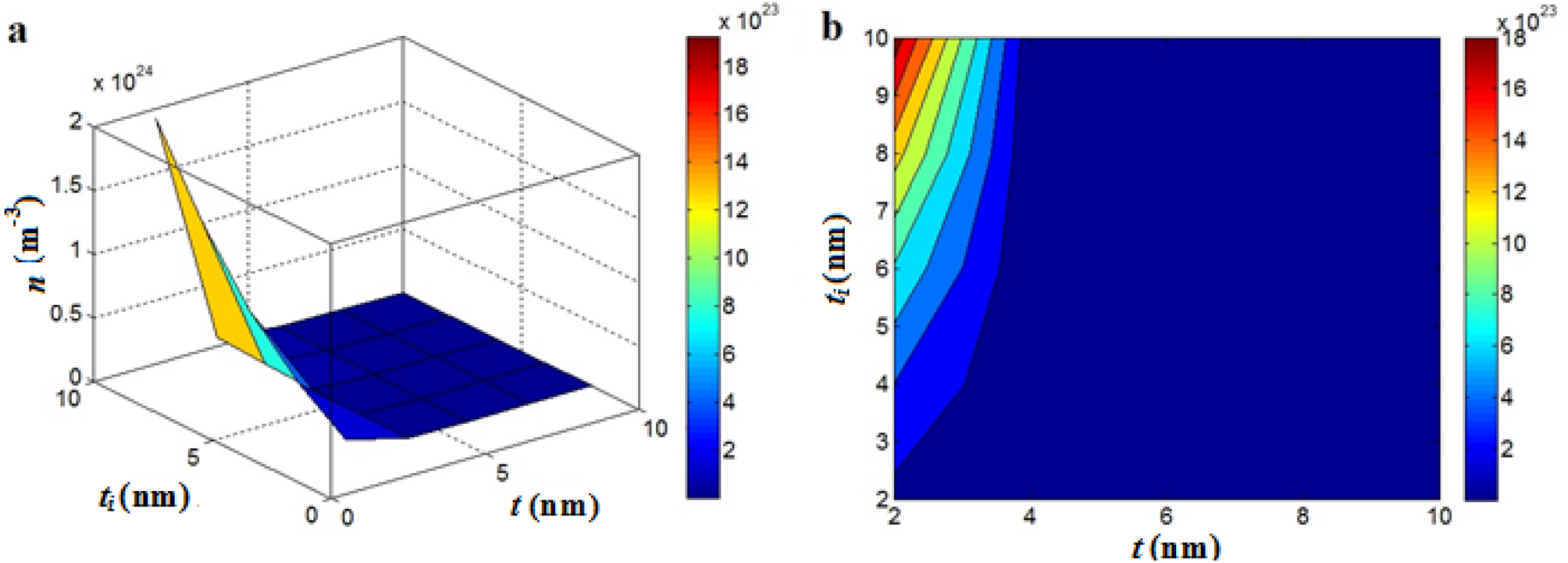
“*n*” linking to “*t*” and “*t*_*i*_” (Eq. 9) at $\phi_{f}$ = 0.01: (**a**) 3D and (**b**) contour plans.

Figure 1 shows the “*n*” level at different values of “*t*” and “*t*_*i*_” in a nanocomposite containing 1 vol % graphene ($\phi_{f}$ = 0.01). The largest number of contacts is gained by the thinnest nanosheets and the densest interphase. As observed, *t* = 2 nm and *t*_*i*_ = 10 nm result in *n* = 18*10^23^, while larger nanosheets than > 4 nm significantly reduce the number of contacts (Fig. 1). Accordingly, thin nanosheets and thick interphase desirably increase the number of contacts. In a constant volume of nanoparticles, thinner ones cause a higher number, which produce more contacts. Also, thick interphase decreases the distance between nanoparticles intensifying their contacts. As a result, Eq. (9) properly shows the impacts of graphene and interphase on the “*n*”.

The tunnel conductivity (Eq. 6) assuming the role of interphase is also expressed as:

10 $$\sigma = \frac{{4\phi_{eff}^{2} Dd}}{{\rho \pi t^{2} }} = \frac{{4\phi_{f}^{2} \left( {1 + \frac{{2t_{i} }}{t}} \right)^{2} Dd}}{{\rho \pi t^{2} }}$$

which ponders the inspirations of tunneling effect and interphase areas on the conductivity. According to Eq. (10), tunnel conductivity of samples depends on graphene concentration, graphene dimensions, interphase depth, tunneling distance and tunnel resistivity.

The percolation onset of haphazardly distributed graphite in nanocomposite was estimated^53^ by:

11 $$\phi_{p} = \frac{{27\pi D^{2} t}}{{4(D + d)^{3} }}$$

However, *D* >> *d* simplifies the latter equation to:

12 $$\phi_{p} = \frac{27\pi t}{{4D}}$$

The interphase parts are made around the both sides of graphene nanosheets. In addition, the tunneling spaces include the distance between two adjacent nanosheets. Therefore, the interphase and tunneling parts can be considered in the last equation as:

13 $$\phi_{p} = \frac{27\pi t}{{4D + 2\left( {\frac{{Dt_{i} + Dd}}{t}} \right)}}$$

which shows the impacts of filler dimension, interphase deepness and tunneling length on the percolation onset. This equation is used to calculate the tunneling length and interphase depth in the samples.

## Results and discussion

### Tested and forecasted conductivity

The innovative model for tunnel conductivity is appraised by the experimented values. The experimental results of graphene-filled samples were selected from previous articles. More details regarding how to fabricate the polymer-graphene nanocomposites are available in the reported references. So, the characterizations or related discussions about the preparation of graphene-based composites are not presented. Four graphene products containing PVA ($\phi_{p}$ = 0.0035, *t* = 2 nm and *D* = 2 μm) from^54^, styrene acrylonitrile (SAN) ($\phi_{p}$ = 0.0017, *t* = 1 nm and *D* = 2 μm) from^22^, polystyrene (PS) ($\phi_{p}$ = 0.0005, *t* = 1 nm and *D* = 4 μm) from^55^ and polystyrene (PS) ($\phi_{p}$ = 0.001, *t* = 1 nm and *D* = 2 μm) from^56^ were chosen for analysis. These samples were fabricated by solution mixing. The real graphene dimensions and percolation onset were reported from the references. However, the values of interphase depth and tunneling distance were not reported in the original articles and these values were obtained by comparing the obtained percolation onset to Eq. (13). Additionally, more information about the raw materials is available in the referred articles.

By comparing the percolation onset of examples to developed Eq. (13), the values of interphase depth and tunneling length are calculated. The (*t*_*i*_*, d*) levels are obtained as (5, 5), (5, 5), (7, 10) and (8, 8) nm for PVA, SAN, PS and PS graphene samples, in that order. The highest levels of interphase depth and tunneling length are detected in PS/graphene examples, since they show very small percolation onset. Actually, a low percolation onset is acquired in a sample with thick interphase and large tunneling length. Accordingly, Eq. (13) can be used to get the percolation onset accounting the roles of interphase and tunneling districts. Moreover, if these terms were not considered, it is not possible to calculate the very slight percolation onset in polymer graphene nanocomposite. It is important to cause a thick interphase and large tunneling length to reduce the percolation onset through controlling the interfacial adhesion and graphene dimensions^57,58^.

**Figure 2:**
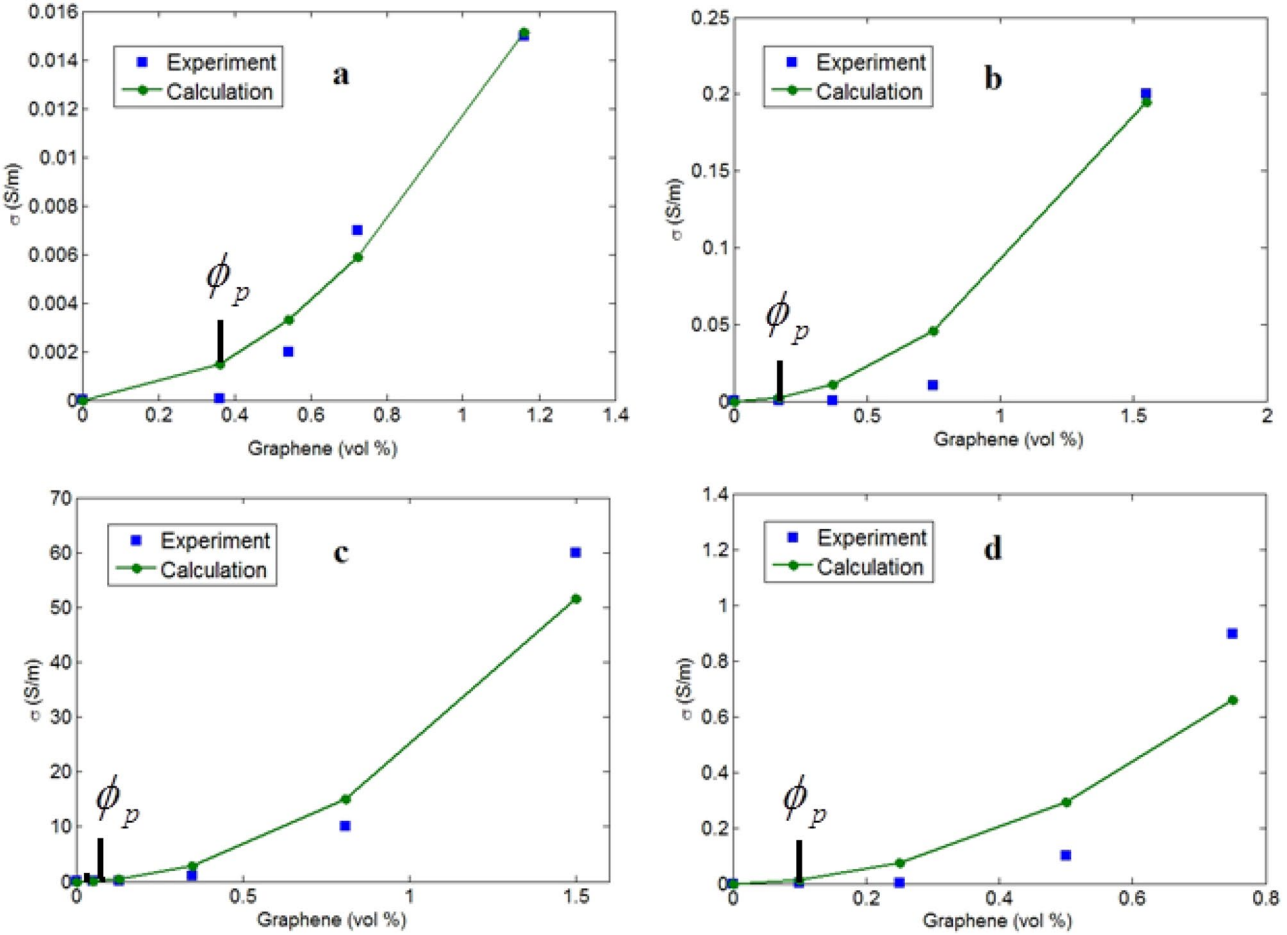
Experimented data and predictions by innovative model for (**a**) PVA^54^, (**b**) SAN^22^, (**c**) PS^55^ and (**d**) PS^56^ graphene examples.

Figure 2 illustrates the experimental and predicted conductivity of the reported samples at different filler amounts using the innovative model (Eq. 10). The estimates suitably follow the experimented points, which demonstrate the predictability of the innovative model. Thus, the novel model based on tunnel conductivity and interphase role can be applied to approximate the conductivity. There are some deviations in calculated values from the experimental values for samples, because some parameters such as aggregation/agglomeration of graphene nanosheets at high concentrations affect the conductivity of nanocomposites. Also, the current model is compared to the experimental facts of various types of polymer matrices (PVA, SAN and PS) and graphene nanosheets and thus this deviation is normal. However, the calculations show good trends with experimental data, which is valuable.

The values of “*ρ*” are obtained as 1020, 1900, 50 and 500 Ω m for PVA, SAN, PS and PS examples, in that order. These data lead to the average intrinsic tunnel resistance and conductivity (*R*_*t*_*, σ*_*t*_) as (1.28, 0.79), (4.75, 0.21), (0.125, 8) and (2, 0.5) (ohm, S) for PVA, SAN, PS and PS examples, correspondingly. So, the smallest “*R*_*t*_” and the highest “*σ*_*t*_” are calculated for PS/graphene nanocomposite (the third sample), while SAN/graphene sample shows the highest tunnel resistance and the lowest tunnel conductivity. The tunnel’s properties significantly change the conductivity, because they govern the level of electron transferring between nanosheets.

### Examination of factors

The effects of all variables on “*R*_*t*_”, “*σ*_*t*_” and “*σ*” as intrinsic tunnel resistance, conductance of tunneling spaces and conductivity are discussed by the presented equations. 3D and contour plots show the variation of results at numerous levels of two factors, but other factors are constant. The variation of parameters and the constant values were selected based on the reported levels in section “Tested and forecasted conductivity” and previous articles. So, the values of all parameters are reasonable and accurate confirming the calculations.

**Figure 3:**
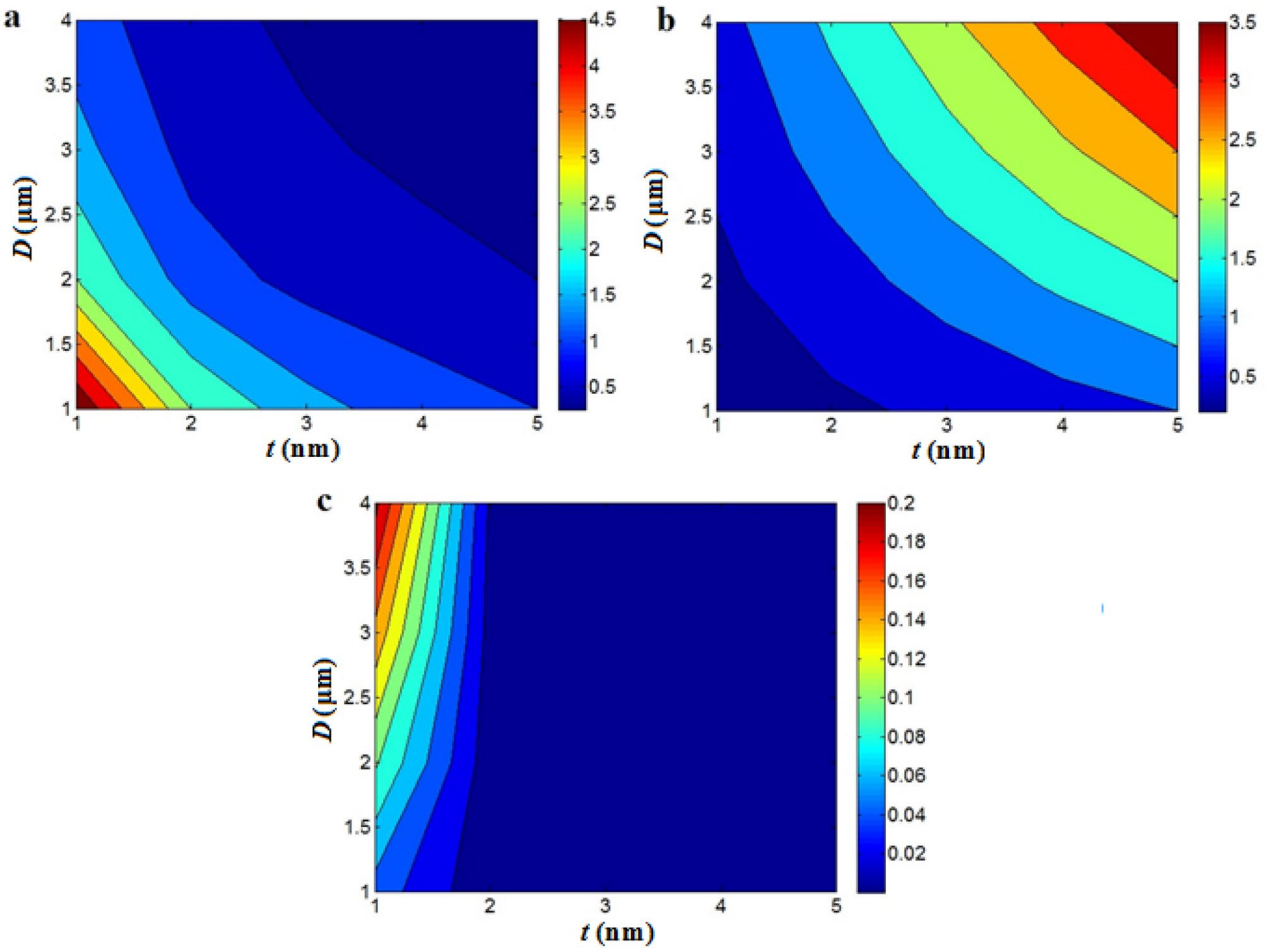
Contour plots for the significances of “*t*” and “D” on (**a**) “*R*_*t*_”, (**b**) “*σ*_*t*_” and (**c**) “*σ*”.

Figure 3 indicates the powers of “*t*” and “*D*” on “R_t_”, “σ_t_” and “σ” at average *d* = 5 nm, *t*_*i*_ = 4 nm, *ρ* = 1000 Ω.m and $\phi_{f}$ = 0.01 by contour plots. The highest “*R*_*t*_” as 4.5 Ω is found at t = 1 nm and *D* = 1 μm, although the high levels of these parameters decrease “*R*_*t*_” (Fig. 3a). In addition, the highest “*σ*_*t*_” as 3.5 S is developed at *t* = 5 nm and *D* = 4 μm, although low *σ*_*t*_ = 0.2 S is witnessed at *t* < 1.8 nm and *D* < 1.5 μm. Thus, the dimensions of nanosheets differently affect the resistance and conductivity of tunneling parts. Thick and long nanosheets obtain the most conductive tunnels, but thin and small nanosheet produce low tunnel conductivity. These evidences are logical, because thick and long nanosheets increase the contact area between adjacent nanosheets, which diminishes the tunnel resistance based on Eq. (4). On the other hand, a big contact area between neighboring conductive nanosheets improves the tunnel conductivity. So, it is expected to acquire a big tunnel conductivity and a little tunnel resistance by thick and large graphene nanosheets.

The best conductivity is achieved at low “*t*” and large “*D*”. As observed in Fig. 3c, the supreme conductivity of 0.2 S/m is calculated at *t* = 1 nm and *D* = 4 μm, while the conductivity weakens to 0 when *t* > 2 nm. It means that the thick nanosheets seriously decrease the conductivity, but thin and large graphene can recover the conductivity. So, the optimistic effects of filler size on the conductivity are obtained by thin and big nanosheets.

Thick nanosheets improve the tunnel conductivity, but they decrease the number of contacts (Fig. 1) and the active amount of nanoparticles (Eq. 8) in the nanocomposite. As a result, thick nanosheets negatively affect the conductivity, because they reduce the number of tunnels and the nanoparticles activeness in the nanocomposite. Generally, thin and big nanosheets can increase the conductivity, because they produce a big aspect ratio, which reduces the percolation onset (Eq. 13) and boosts the net size. Moreover, thin nanosheets increase the number of contacts and volume share of interphase parts (Eq. 7) in the nanocomposite, which directly manage the conductivity. Really, thin and big nanosheets highlight the influences of nanoparticles as super-conductive phase on the conductivity via the growth of contacts between nanoparticles and enhancement of interphase areas. Therefore, it is judicious to show a high conductivity by skinny and large nanosheets, although thick nanosheets can produce strong tunnel conductivity. By these reasons, the innovative model appropriately suggests the roles of graphene dimensions in the conductivity.

**Figure 4:**
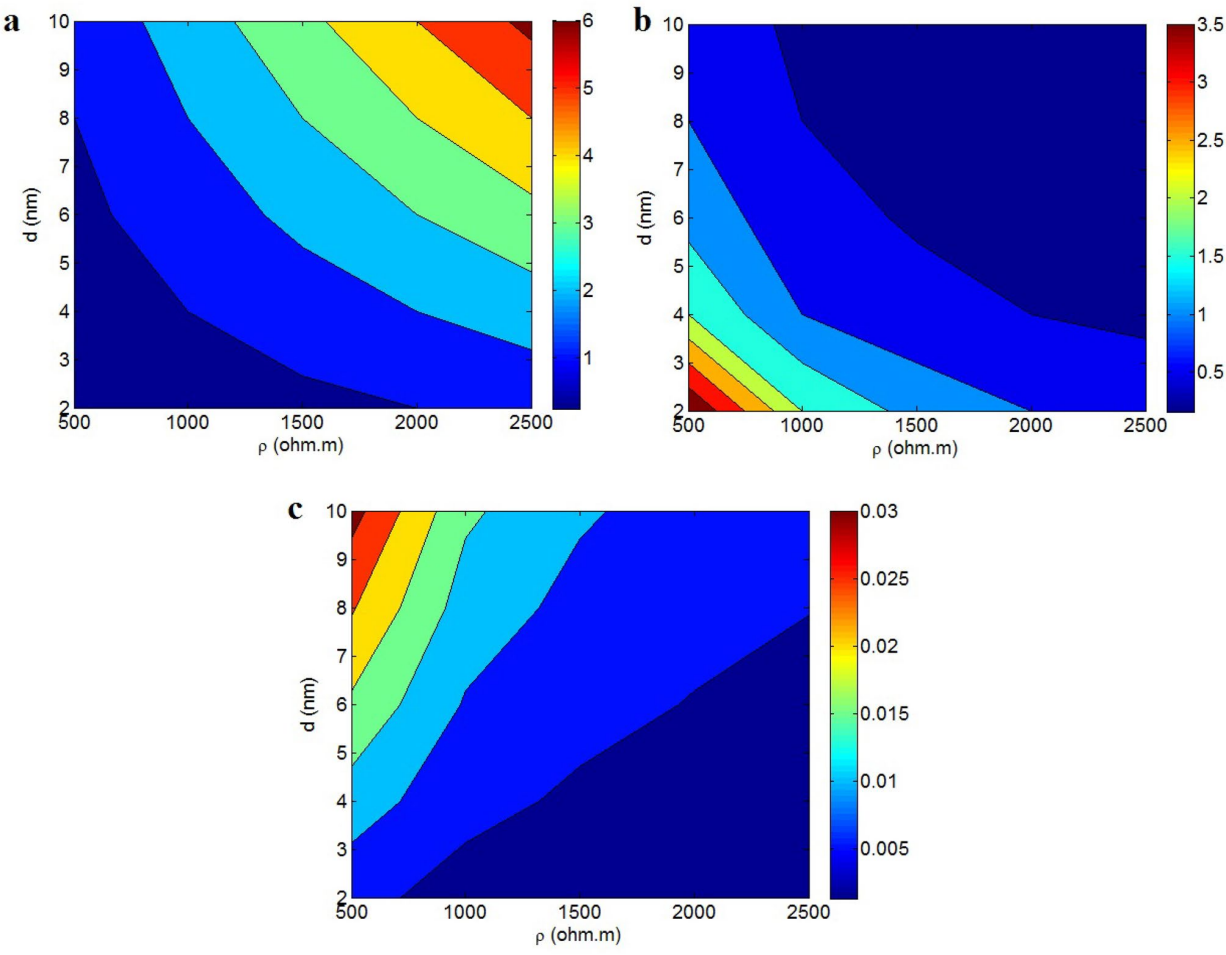
Impresses of “*ρ*” and “*d*” on (**a**) “*R*_*t*_”, (**b**) “*σ*_*t*_” and (**c**) “*σ*” at *D* = 2 μm, *t* = 2 nm, *t*_*i*_ = 4 nm and $\phi_{f}$ = 0.01.

The dependencies of “*R*_*t*_”, “*σ*_*t*_” and “*σ*” on “*ρ*” and “*d*” are shown in Fig. 4 at *t* = 2 nm, *D* = 2 μm, *t*_*i*_ = 4 nm and $\phi_{f}$ = 0.01. As revealed, *ρ* = 2500 Ω m and *d* = 10 nm result in the highest tunnel resistance of 6 Ω, while little extents of these factors significantly fall the tunnel resistance. In addition, the maximum tunnel conductivity of 3.5 S is obtained at *ρ* = 500 Ω m and *d* = 2 nm, while *ρ* > 1500 Ω.m and *d* > 6 nm decrease the tunnel conductivity to about 0.1 S. Consequently, small tunnel resistivity and short tunnels can improve the tunnel conductivity and decrease its intrinsic resistance. These evidences are logical, since the resistivity of tunnels directly change the tunnel resistance based on Eq. (4). On the other hand, a low distance between nanosheets increases the tunnel conductivity, because the inter-particle spaces commonly contain the insulated polymer matrices. Actually, a short tunneling length reduces the effect of insulated polymer and grows the roles of conductive graphene nanosheets and their interactions. So, it is practical to find a low resistance and good conductivity in small tunneling parts.

Figure 4c reveals the tunnel conductivity at different “*ρ*” and “*d*” levels. The conductivity grows to 0.03 S/m at *ρ* = 500 Ω m and *d* = 10 nm, while it approaches to 0 when *ρ* > 1500 Ω m and *d* < 6 nm. Accordingly, the desirable conductivity is achieved by low tunnel resistivity and large tunnels. Both the conductivity of tunnels and whole nanocomposite show high levels at small values of “*ρ*”, but they display dissimilar levels at different “*d*”.

As mentioned, a great tunnel conductivity is gained by short tunnels, but the conductivity directly links to tunneling length. According to Eq. (10), the conductivity is directly correlated to the tunneling length, while “d” inversely controls the tunnel conductivity (Eq. 5). Although a large tunneling length shows a poor intrinsic conductivity, but it can participate many nanosheets to the conductive networks to increase their size and density. In addition, large tunneling length can quicken the percolation onset to produce the conductive networks (Eq. 13). So, a large tunneling length positively affects the conductivity by lessening of percolation onset and enhancement of networks extents. Also, a high “ρ” poorly transfers the electrons between adjacent nanosheets weakening the conductivity. Thus, little “ρ” and long tunnels can definitely control the tunnel conductivity through the formation of big and active networks. However, it was reported that the tunneling length has a maximum about 10 nm in polymer CNT nanocomposite^59^. It can be suggested that the neighboring nanosheets far from than 10 nm cannot show the tunneling effect, which do not change the conductivity.

**Figure 5:**
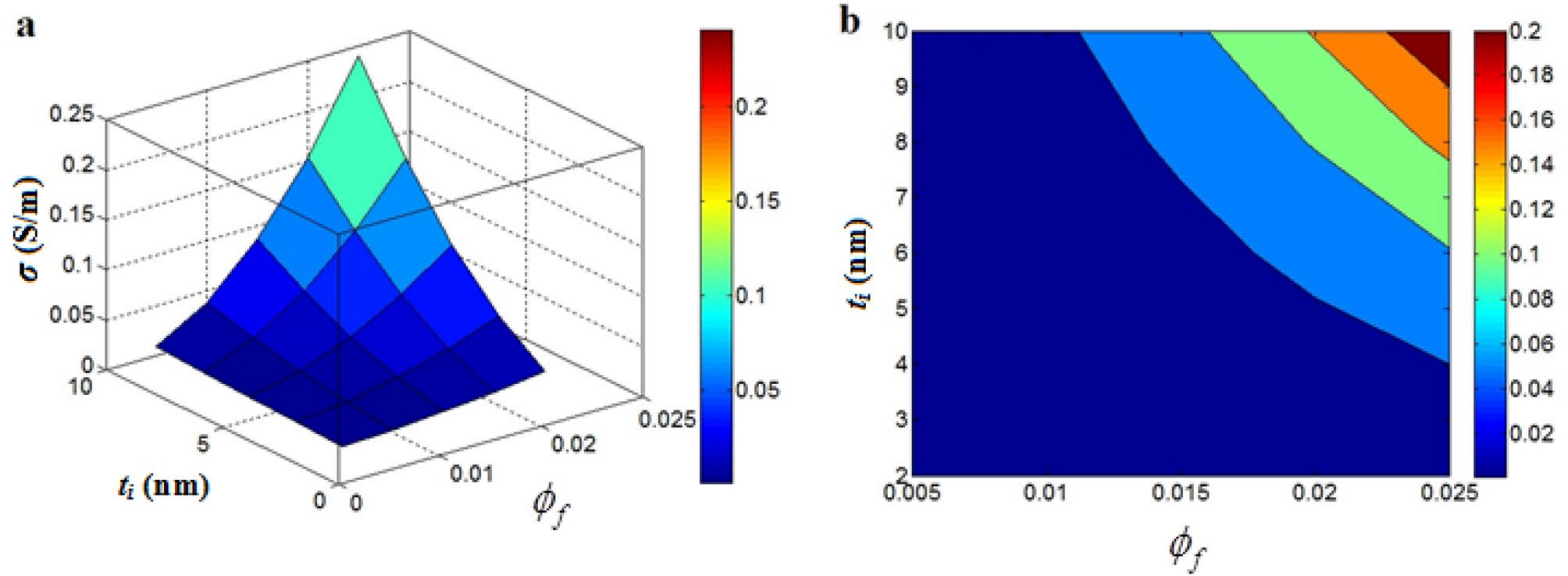
(**a**) 3D and (**b**) contour images for associations of conductivity to “$\phi_{f}$” and “*t*_*i*_”.

Figure 5 also shows the stimuli of filler amount and interphase depth on the conductivity at *d* = 5 nm, *t* = 2 nm, *D* = 2 μm and ρ = 1000 Ω m. The best conductivity of 0.2 S/m is obtained at $\phi_{f}$ = 0.025 and *t*_*i*_ = 10 nm, although the slight values of these parameters as $\phi_{f}$ < 0.015 and *t*_*i*_ < 7 nm produce an insulated nanocomposite. So, filler quantity and interphase depth rightly govern the conductivity.

The positive influence of filler amount on the conductivity is interpreted by the more significant conductivity of graphene than polymer mediums^60^. In fact, the conductivity is controlled by the conductivity and amount of conductive graphene, because the major phase of polymer matrix is insulated. The straight link among conductivity and filler share was observed in many experimental and theoretical articles^61,62^, but the conductivity becomes relatively constant at high filler amounts^27^. In addition, thick interphase can produce large and dense conductive networks in the nanocomposite, because they take part in the percolation occurrence and network structures. In fact, big interphase parts can show a pseudo-percolation at low filler shares, due to their attachments in the nanocomposite. The confident character of interphase depth in the percolation onset was stated in many works^63,64^, nevertheless its impression on the conductivity was not explored. Based on the mentioned remarks, the new model appositely states the right dependences of conductivity on filler amount and interphase depth.

## Conclusions

Several simple equations were expressed for tunnel conductivity, tunnel resistance and whole conductivity by the number of contacts and interphase parts. The conductivity was suggested by filler share, filler dimensions, tunneling length and interphase depth. The estimates of the innovative model agree with the tentative data of real examples and the effects of terms on the tunnel resistance, tunnel conductivity and conductivity are judicious, which justify the developed equations. Thin and big nanosheets positively improve the conductivity, but thick nanosheets can increase the tunnel conductivity. The thick nanosheets increase the conductivity of tunnels, but they decrease the number of tunnels and the active amount of nanoparticles. In addition, big tunnel conductivity is obtained by short tunnels, but the conductivity directly connects to the tunneling length, since large tunnels can quicken the percolation onset, push many nanosheets to the nets and increase the size and compactness of nets. So, a large tunneling length generally improves the conductivity. Furthermore, both filler amount and interphase depth straightly govern the conductivity, due to the optimistic contributions of graphene and interphase parts in the performances of nets.

## Data Availability

The data that support the findings of this study are available on request from corresponding author.
